# Publisher Correction: Maritime Continent water cycle regulates low-latitude chokepoint of global ocean circulation

**DOI:** 10.1038/s41467-019-10791-z

**Published:** 2019-06-21

**Authors:** Tong Lee, Séverine Fournier, Arnold L. Gordon, Janet Sprintall

**Affiliations:** 1 Jet Propulsion Laboratory, California Institute of Technology, Pasadena, CA 91011 USA; 2 Lamont-Doherty Earth Observatory, Columbia University, Palisades, NY 10964 USA; 3 Scripps Institution of Oceanography, University of California, San Diego, CA 92037 USA

**Keywords:** Hydrology, Physical oceanography

Correction to: *Nature Communications* 10.1038/s41467-019-10109-z, published online 08 May 2019.

The original version of this Article contained errors in Figs. 7 and 8 in which the y-axes labels were omitted. In Fig. 7, the y-axes labels on both panels now read ‘SSS tendency (pss/month)’. In Fig. 8, the y-axes labels on both panels now read ‘Horizontal advective tendency (pss/month)’.

In the original version of Fig. 10, the title incorrectly read ‘Meridional SSS profile along Makassar Strait’. The correct version replaces ‘SSS’ with ‘SLA’.

Finally, the second sentence of the fourth paragraph of the ‘Data’ section of the Methods originally incorrectly read ‘The product is available through https://podaac.jpl.nasa.gov/dataset/OSTIA-UKMO-L4-GLOB-v2.0.’ The correct version replaced the web link with ‘https://www.catds.fr/Products/Available-products-from-CEC-OS/CEC-Locean-L3-Debiased-v3’.

This has been corrected in both the PDF and HTML versions of the Article.

